# *S*-Adenosine Methionine (SAMe) and Valproic Acid (VPA) as Epigenetic Modulators: Special Emphasis on their Interactions Affecting Nervous Tissue during Pregnancy

**DOI:** 10.3390/ijms21103721

**Published:** 2020-05-25

**Authors:** Asher Ornoy, Maria Becker, Liza Weinstein-Fudim, Zivanit Ergaz

**Affiliations:** 1 Medical Neurobiology Department, Hebrew University Hadassah Medical School, Jerusalem 91120, Israel; liza.weinstein-f@mail.huji.ac.il (L.W.-F.); Zivanit@hadassah.org.il (Z.E.); 2 Institute for Translational Research, Adelson School of Medicine, Ariel University, Ariel 40700, Israel; mariabe@ariel.ac.il

**Keywords:** S-adenosyl methionine, valproic acid, epigenetic modifications, gene expression, brain, pregnancy, methylation, demethylation, nervous system

## Abstract

*S*-adenosylmethionine (SAMe) is involved in many transmethylation reactions in most living organisms and is also required in the synthesis of several substances such as monoamine neurotransmitters and the N-methyl-D-aspartate (NMDA) receptor. Due to its important role as an epigenetic modulator, we discuss in some length the process of DNA methylation and demethylation and the critical periods of epigenetic modifications in the embryo, fetus, and thereafter. We also discuss the effects of SAMe deficiency and the attempts to use SAMe for therapeutic purposes such as the treatment of major depressive disorder, Alzheimer disease, and other neuropsychiatric disorders. SAMe is an approved food additive and as such is also used during pregnancy. Yet, there seems to scanty data on the possible effects of SAMe on the developing embryo and fetus. Valproic acid (VPA) is a well-tolerated and effective antiepileptic drug that is also used as a mood stabilizer. Due to its high teratogenicity, it is contraindicated in pregnancy. A major mechanism of its action is histone deacetylase inhibition, and therefore, it acts as an epigenetic modulator, mainly on the brain. This prompted clinical trials using VPA for additional indications i.e., treating degenerative brain disease such as Alzheimer disease, dementia, HIV, and even cancer. Therefore, we discuss the possible effects of VPA and SAMe on the conceptus and early postnatally, during periods of susceptibility to epigenetic modifications. VPA is also used as an inducer of autistic-like behavior in rodents and was found by us to modify gene expression when administered during the first postnatal week but not when administered to the pregnant dams on day 12 of gestation. In contrast, SAMe modified gene expression when administered on day 12 of pregnancy but not postnatally. If administered together, VPA prevented the changes in gene expression induced by prenatal SAMe administration, and SAMe prevented the gene expression changes and autistic-like behavior induced by early postnatal VPA. It is concluded that both VPA and SAMe are powerful epigenetic modifiers with antagonistic actions on the brain that will probably be used in the future more extensively for the treatment of a variety of epigenetic diseases of the nervous system.

## 1. Introduction

Epigenetic mechanisms are currently related not only to a variety of biological and developmental processes but also in the pathogenetic mechanisms of a variety of congenital malformations and diseases, including many neurobehavioral and psychiatric disorders [1,2]. Moreover, various epigenetic modulators are being offered for possible therapy of "epigenetic diseases" [2]. Among them are S-adenosylmethionine (SAMe) and valproic acid (VPA) as they are involved in DNA methylation/demethylation. In this review, we will specifically discuss the effects of these substances in a variety of biological processes with special emphasis on their effects during pregnancy. In addition, due to their antagonistic effects on DNA methylation, we will discuss the current data on their possible interaction while administered together.

VPA is a well-known, well-tolerated antiepileptic drug and mood stabilizer that is widely used for the treatment of epilepsy, especially in children. Since VPA is a proven human teratogen, apparently the most teratogenic agents among the antiepileptic drugs, it is generally contraindicated in women at child-bearing age except in conditions where VPA is the drug of choice. In the last years VPA, mainly due to its action as a histone deacetylase inhibitor, is used in a variety of other diseases such as Alzheimer disease, HIV, and cancer. 

SAMe, an FDA-approved food additive sold without prescriptions (OTC) is also being recommended for use in a variety of psychiatric and neurological diseases such as bipolar disorder, depression, and Alzheimer disease. Thus, despite being a methyl donor with antagonistic action to those of VPA, both are often recommended for use for similar psychiatric indications. Compared to the known teratogenic effect of VPA, there is very little data regarding the possible effects of SAMe on pregnancy and pregnancy outcome.

In this review, we will discuss the effects of both VPA and SAM as epigenetic modulators and the mechanisms underlying their effects on the epigenome, with special emphasis on pregnancy. We will also discuss their effects when administered together in relation to autism spectrum disorder (ASD) and other diseases that are associated with epigenetic modification.

### S-Adenosylmethionine: The Principal Physiological Methyl Donor

S-adenosylmethionine (SAM, also known as AdoMet and SAMe) is an active metabolite because it has a chemically reactive methyl group (CH3) and is involved in many physiological transmethylation reactions in all living organisms [3,4]. Being the main biological methyl donor, SAMe is used as a precursor for the synthesis of polyamines, lipids, nucleotides, proteins, monoamine neurotransmitters, and glutathione (GSH) via three main interconnected metabolic pathways: polyamine synthesis, transmethylation, and transsulfuration [3,4].

To provide the maintenance of the normal function of biochemical processes in the body, a balanced amount of SAMe is required. SAMe levels depend on methionine availability, obtained from the diet and its production by de novo synthesis in the presence of methyltetrahydrofolate and vitamin B12 [5]. SAMe is essential for normal biological functions and development, and its deficit can lead to pathological changes, particularly in the central nervous system (CNS). 

The regulatory role of SAMe in the synthesis and function of monoamine neurotransmitters (such as noradrenaline, adrenaline, dopamine, serotonin, and histamine) was demonstrated in several studies. It was shown that administration of substrates such as L-dihydroxyphenylalanine, which facilitates dopamine synthesis, led to a prominent decrease in SAMe concentrations in the brains of rats [6], and in whole blood [7] and cerebrospinal fluid (CSF) [8] in humans. Moreover, SAMe concentrations were also reduced in rat brain tissues treated with monoamine reuptake inhibitors [9].

SAMe is also involved in the synthesis of the N-methyl-D-aspartate (NMDA) receptor via the transamino–propylation pathway [3] and in the transformation of norepinephrine to epinephrine and polyamine, which modulates NMDA receptor excitability [10]. Thus, SAMe also contributes to the homeostasis of monoamine levels in brain tissues. 

**Figure 1:**
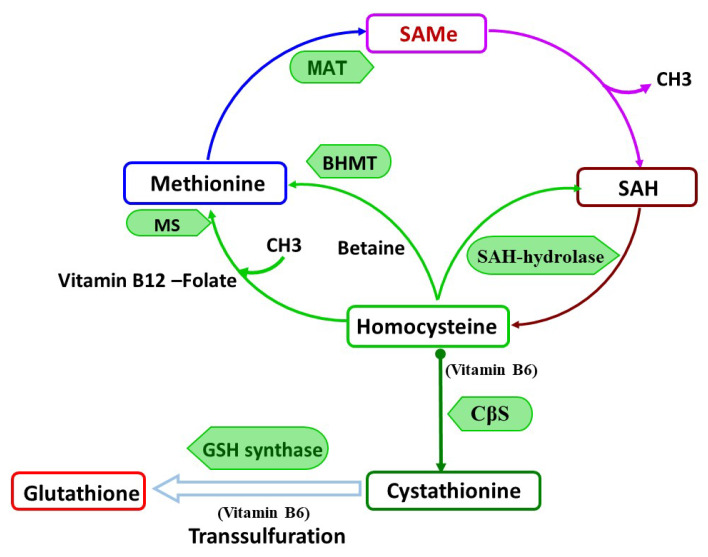
*S*-adenosylmethionine (SAMe) metabolism.

SAMe is formed in all cells of the body. However, in the liver, which is the major site for SAMe metabolism [9], nearly 50% of the daily intake of methionine is converted to SAMe and nearly 85% of all methylation reactions occur, whereas in the brain only approximately 20% of the daily intake of methionine is utilized [11]. In healthy human, about 6–8 g of SAMe is generated per day and used in different transmethylation reactions. The active methyl groups from SAMe are transmitted to other molecules resulting in the formation of S-adenosylhomocysteine (SAH) [3,9]. The balance between SAMe and SAH (SAMe to SAH ratio) is a key factor in the regulatory control of all transmethylation reactions [4] (Figure 1). Thus, the increase in SAH level or a decrease in the SAMe level could lead to the inhibition of transmethylation reactions [4] and affect numerous cellular processes, such as DNA methylation and gene expression [9]. 

DNA methylation is the process of the methyl group transfer from S-adenosylmethionine to cytosine usually in the fifth position (5methylcytosine, or 5mC) of the cytosine residue in CpG dinucleotides, which are commonly found in promoter regions [12,13]. DNA methylation, particularly in CpG islands (CGIs) and gene promotor regions, causes the repression of gene transcription, as found in the inactivated X chromosome in females. However, in unsilenced genes, the higher levels of methylation are within gene bodies on both the active X and inactivated X, and reduced promoter methylation is only on the inactivated X chromosome [14,15].

## 2. Overview of DNA Methylation and Chromatin Modification Machinery

Epigenetic changes triggered by various extrinsic factors during prenatal and early postnatal life can affect the normal physiological and metabolic functions at adulthood and may be prediction risk factors for disease development [15,16]. Epigenetic changes are determined by the processes that regulate patterns of gene expression, such as DNA methylation, and post-translational modifications of the nucleosome core histones, which lead to the altered packaging of DNA and the chromatin structure, while the genomic DNA sequence remains preserved [12]. These processes, which regulate patterns of gene expression, use the proteins that add, remove, or interpret the modified DNA or histones, and are referred as “writers”, “erasers”, and “readers”. 

The transfer of the active methyl group from SAMe to cytosine is carried out by DNA methyltransferases (DNMTs) [17]. In mammals, there are three enzymatically active DNMTs, the DNMT1, DNMT3A, DNMT3B, and one acting as stimulatory factor, DNMT3L, which are large proteins with differences in a catalytic C-terminal domain and in a regulatory N-terminal part [17]. During the embryonic development of mammals, DNA methylations are mediated by DNMT3A and DNMT3B, with the assistance of the DNMT3L. DNMT3A and DNMT3B are considered as de novo methyltransferases [18] and in adults are presented at high levels mainly in undifferentiated cells and germ cell precursors, whereas their levels in somatic cells are significantly low. 

The established DNA methylation patterns during early development are preserved for the rest of the life by methyltransferase DNMT1, which is considered as a maintenance methyltransferase [19,20,21]. The DNA methylation or demethylation processes are dependent on the levels of SAMe. 

5 methyl cytosine (5mC) can be recognized and interpreted by methyl-CpG-binding proteins, referred as “readers”, which have the methyl-binding domain (MBD). The main “reader” is a methyl CpG binding protein 2 (MeCP2), which recognizes 5mC, a methylation tag, and further recruits chromatin remodeling complexes [22], such as histone acetylation and histone methylation [23,24]. Therefore, DNA methylation tags promote the certain histone states by the activation of machinery of post-translational histone modifications (PSMs) including acetylation, methylation, and ubiquitination [25]. Of the many existing histone modifications, the histone acetylation of ϵ-amino groups on lysine residues in H3 and H4 tails is associated with promoting gene transcription [25,26], whereas the deacetylation of histones leads to the repression of gene transcription. The histone acetylation process is controlled by the antagonistic actions of two large families of enzymes: the histone acetyltransferases (HATs) and the histone deacetylases (HDACs) (reviewed in [27]).

Demethylation processes are carried out by “erasers”, ten-eleven translocation (TET) dioxygenases (TET1, TET2, and TET3), which cause a sequence of oxidation reactions transforming the 5-methylcytosine (5mC) initially to 5-hydroxymethylcytosine (5hmC) and then to a 5-formyl-cytosine (5fC) and a 5-carboxyl-cytosine (5caC) [28,29]. The last transforms, 5fC and 5caC, may be converted to demethylated C by terminal deoxynucleotidyl transferase (TdT). 

### 2.1. Sensitive Periods for Epigenetic Changes

The critical period (CP) refers to a narrow developmental window when the system is especially sensitive to environmental stimuli and produces permanent changes to neural circuits. [30]. It is generally considered that major rounds of epigenetic reprogramming occur during early embryonic development, soon after fertilization [12,31]. In order to ensure further tuning of the pluripotent ground condition for the early embryo, most 5mC and 5hmC are demethylated (“erased”). Then, de-novo methylation events occur by finely tuned epigenetic machinery during the prenatal stage, forming the further epigenetic pattern [31]. Around the time that the blastula implants into the uterus, there is an increase of activity in DNMT3A/B enzymes and TET1 and TET2 with a simultaneous increase in 5hmC in the inner cell mass [31]. Therefore, the impaired function of DNMTs or TET dioxygenases may compromise normal embryonic development. In the study using neomycin conditional knockout DNMT3A mice, it was shown that the embryonic deletion of DNMT3A results in neuronal dysfunction, which is associated with hypoactivity, motor abnormalities, fewer motor neurons, and a shortened lifespan [32]. Another study carried out in the model of Tet Triple-Knockout mice demonstrated that the depletion of all forms of TETs restricted cell lineage differentiation and caused aberrant embryonic development [33]. 

The analysis of multiple isogenic organ sets showed that organ-specific DNA methylation patterns were highly dynamic in the human embryo between week 9 (W9) and W22 of gestation [34], at gestational ages when different immature organ systems are sensitive to functional defects. In addition, a large percentage of the tissue-specific methylation pattern was found to be generated also during the early postnatal period [35]. 

The third round of epigenetic reprogramming occurs at puberty, which is a period that is characterized by shifts in male and female sex steroid hormone production. This transition from child to adult phenotype is initiated by central activation of the hypothalamic–pituitary–gonadal (HPG) axis, resulting in the stimulation of steroid hormone synthesis, and it is reflected by changes in the patterns of DNA methylation observed in peripheral white blood cells in humans [36] and in the pituitary gland of mice [37].

### 2.2. Differences and Similarities in Critical Periods between Rodents and Humans

The sequence of events in the developing embryo and fetus might be comparable among species, although the time scales are greatly different [35,38]. The nervous system development in rodents has considerable postnatal development, whereas humans undergo more prenatal maturation of their brain [39]. This has important meanings from the aspect of the morphological and functional analysis of the timing of exposure to various environmental stimuli and epigenetic changes that may be manifested later in life. Thus, the epigenetic changes during Gestational Day (GD) 9–9.5 in mice and GD 20–21 in humans could be expected to interfere with neural tube formation, when the epigenetic changes during week 4 of gestation in humans (or on GD 10 in mice) would be expected to interfere with neurogenesis in the spinal cord and hindbrain structures. Hence, epigenetic changes at the end of gestation in humans or in the first postnatal week in mice might affect neurogenesis in the cerebellum and hippocampus, and probably other ongoing processes such as cell migration, myelination, and synaptogenesis, in areas that have already undergone neurogenesis [35,38,40].

### 2.3. Methyl Donors and Major Depression Disorder (MDD)

Agents that may modify the epigenome (SAMe, methionine, choline, B12, folic acid, and betaine) were studied in clinical trials in major depression disorder (MDD), schizophrenia, and bipolar depression syndrome [41,42,43].

Since SAMe is a main methyl donor for many physiological processes, it has been proposed as a potential treatment agent for many diseases, particularly MDD [44], primary and secondary fibromyalgia [44,45], attention-deficit hyperactivity disorder [31], Parkinson’s disease [32], senile dementia, and Alzheimer disease [7]. The beneficial effects of SAMe were also reported in the treatment of osteoarthritis, where SAMe demonstrated some anti-inflammatory effect, resembling nonsteroidal anti-inflammatory drugs [46]. The most investigated and prominent therapeutic effect of SAMe is in alcoholic hepatitis and cirrhosis [47,48,49]. 

Treatment with SAMe as monotherapy for MDD demonstrated significant efficacy and good tolerability [50,51]. Moreover, it was shown that SAMe used for adjunct treatment accelerated and enhanced the action of tricyclic antidepressants and Selective Serotonin Reuptake Inhibitor SSRIs in poor responders MDD patients [42,44,50,51,52]. These clinical reports are well correlated with broadly published data, which described altered gene expression and decreased levels of methylation in samples derived from depressive patients and from animal models of psychiatric disorders [52,53,54,55]. It was also shown that SAMe administered to mice before exposure to stressful stimuli such as a forced swim test (FST) alleviated depression-like behavior. This was manifested in reduced immobility response 24 h later, enhanced CpG methylation at the immediate–early gene (IEG) and the suppression of IEG expression in the granule neurons of the dentate gyrus [56]. 

It has been reported that inflammation plays an important role in the etiology of depression [57,58]. SAMe reduced the expression of the pro-inflammatory cytokine interleukin 6 (IL6) [59], and it increased the expression of the anti-inflammatory cytokine interleukin 10 (IL10) [60] in lipopolysaccharides (LPS)-stimulated monocytes/macrophages. In addition, SAMe led to altered methylation capacity and blocked the binding of trimethylated H3K4 to the Tumour Necrosis Factor alpha (TNF-α) promoter through increasing methylthioadenosine (MTA) and SAH levels [61].

### 2.4. Homocysteine as a Possible Marker of SAMe Deficiency and Epigenetic Changes

Homocysteine (HCY), a sulfurated amino acid, is formed in methionine turnover by the conversion of SAH in to homocysteine and adenosine by SAH hydrolase (SAHH) [5] (see Figure 1). Nearly 50% of formed HCY is further converted to cysteine by the transulfuration pathway [3,4] for further glutathione formation. It was shown that the SAMe intracellular level is a modulator factor, that controls by which pathway either re-methylation or transsulfuration, homocysteine will be eliminated [3]. Thus, higher levels of SAMe facilitate the transsulfuration pathway and therefore glutathione formation. However, SAMe deficiency cause a shift to homocysteine remethylation and de novo methionine formation in the presence of vitamin B12 and folate as a co-factors [3,5,62]. Moreover, the SAH hydrolase is a reversible enzyme that was prompted to re-form SAH in the case of elevated HCY levels and therefore affect the SAMe/SAH ratio, causing the inhibition of all methylation reactions [3]. Thus, a glitch in any step in a methionine turnover, due to dietary deficiency in methionine, vitamin 6, vitamin B12, or folate, may cause a vicious circle that can contribute to the development of many diseases states.

Figure 1 describes SAMe metabolism and the methylation cycle: S-adenosyl methionine (SAMe) is formed by methionine conversion by methionine adenosyltransferase (MAT). When SAMe donates its methyl group during transmethylation reactions, it is converted into S-adenosylhomocysteine (SAH). Furthermore, SAH is catalyzed by SAH hydrolase to form homocysteine. This hydrolysis is a reversible process, dependent on the SAMe/SAH ratio and further homocysteine metabolism. Homocysteine can undergo three metabolic reaction: two remethylation pathways resynthesize methionine (folate/vitamin B12-dependent) and betaine, (the choline metabolite dependent that is catabolized by betaine–homocysteine methyltransferase), and the transsulfuration pathway, which require vitamin B6 as a co-factor, resulting in glutathione formation. MS: Methyl transferase; SAM: Methionine adenosyltransferase SAMe: S-adenosylmethionine; SAH: S-adenosylhomocysteine; SAH-hydrolase: S-adenosylhomocysteine hydrolase; CβS: Cystathionine β-synthase, GSH synthase-Glutathione synthase; BHMT: betaine-homocysteine methyltransferase.

The accumulation of HCY increases the production of reactive oxygen species (ROS), diminishes antioxidant defense, promotes oxidative stress and mitochondrial dysfunction, and induces apoptosis in rats [7]. It was also reported that in human, elevated levels of HCY may cause the hypomethylation or hypermethylation of cellular DNA and further DNA fragmentation, which may activate cellular apoptosis in the brain [8].

HCY in the plasma is presented in several forms, where the protein-bound total HCY (S-linked, and N-linked) is mostly abundant [63]. Total HCY (tHCY) is used as a predictive risk factor for cardiovascular disorders, stroke progression, screening for inborn errors of methionine metabolism, and as a supplementary test for vitamin B12 deficiency [64]. Moreover, in older depressed patients, high level of plasma HCY may indicate a risk factor for impaired memory and cognitive performance [65].

tHCY was suggested as a sensitive marker of functional deficiency of folic acid and vitamin B12 in patients with MDD [66]. In addition, the plasma folate deficiency positively correlated with a high plasma HCY and a significant low SAMe level and monoamine metabolites, such as 5-hydroxyindoleacetic acid (5HIAA, the main metabolite of serotonin), homovanillic acid (HVA, a major catecholamine metabolite), and 3-methoxy-4-hydroxyphenylglycol (MHPG, a metabolite of norepinephrine degradation) levels in the CSF [66]. It was reported that depressive patients with folate deficiency or high HCY plasma level are poor responders to antidepressant treatment [67,68].

### 2.5. Oxidative Stress in MDD, ASD, and Alzheimer Disease and the Effects of SAMe

Oxidative stress is considered as a shift in the balance between formation of highly reactive molecules, reactive oxygen species (ROS), and the reactive nitrogen species (RNS), and their elimination by antioxidant defense mechanisms [69]. Glutathione (GSH) is the most abundant non-enzymatic endogenous antioxidant. Glutathione formation is highly dependent on methionine availability and its conversion into SAMe, which further facilitates the transsulfuration pathway [4,5,9], resulting in homocysteine conversion into cysteine, in the presence of vitamin B6. 

Oxidative stress has been implicated in the pathogenesis of neurologic and psychiatric diseases [70,71,72]. The brain is considered as particularly vulnerable to oxidative damage due to its high oxygen consumption, low antioxidant defense, and large amounts of oxidation-sensitive lipids [73,74]. Studies have shown that the brains of MDD and anxiety patients are under increased oxidative stress as a result of mitochondrial dysfunction [75,76,77]. Some studies have demonstrated in depressed patients abnormal oxidative product levels in the peripheral blood [78,79], red blood cells (RBC) [79,80], mononuclear cells [79], urine [81], cerebrospinal fluid [82], and postmortem brains [82]. In addition, lower GSH levels [83] and markers of increased oxidative stress [84] were observed in erythrocytes and in postmortem brain of individuals with ASD [85,86]. 

Many animal studies demonstrated the beneficial effect of polyphenols, natural antioxidant compounds found in plant-based foods, in depressive-like [87,88,89] and in ASD-like [90] behavior. In a rat model of depression induced by corticosterone administration, treatment with curcumin, a known potent plant antioxidant, significantly suppressed depression-like behavior and delayed the deterioration of brain Brain-derived neurotrophic factor (BDNF) levels [87]. Moreover, it was reported that curcumin inhibited LSD1 enzymatic activity in vitro [91], and this effect was not dependent on its anti-oxidative properties. This evidence supports the notion that behavioral alterations in depression may arise from the enhanced brain oxidative stress, which is regulated by DNA methylation and histone modifications. 

Green tea extract, an important dietary source of polyphenols, particularly flavonoids, attenuated autistic-like behavior and abnormal nociception in mice postnatally exposed to VPA. The histological analysis has demonstrated that green tee extract protected cerebellar Purkinje cells against VPA damage [90]. Similarly, Piperine, a major alkaloid of the black pepper, also was tested in an autistic-like model induced by treatment with VPA postnatally at day 14 to BALB/c mice [92]. Piperine also normalized the autistic-like behavior, protected neurons in the cerebellum, and in addition, elevated glutathione levels and reduced the ROS in the brain in a dose-dependent manner.

Another antioxidant, N acetylcysteine (NAC), a precursor of glutathione and a detoxifying agent that is used in clinical practice as antidote to acetaminophen intoxication and chronic obstructive pulmonary disease, was recently used as an add-on treatment for bipolar disorder and MDD [93,94,95]. However, it was reported that NAC did not inhibit the LSD1 enzymatic activity [91], and its antidepressant effect was due to glutathione activity.

There are only a few studies on the possible antioxidant effects of SAMe as a protecting agent in psychiatric and neurological diseases. Lieber et al. demonstrated in 1990 [96] the beneficial effects of SAMe on alcohol-induced liver disease in baboons [97]. Later, human studies have shown the beneficial effects of SAMe in liver diseases, demonstrating that SAMe supplementation restored hepatic glutathione (GSH) deposits and attenuated liver injuries such as alcohol liver disease or viral cirrhosis [96,97,98]. 

Several studies investigated the possible effects of SAMe as an antioxidant in animal models of Alzheimer disease. Fuso et al. [99] induced Alzheimer-like brain lesions in TgCRND8 mice by vitamin B-deficient diets. Among the changes were amyloid deposition and an increased expression of Presenilin-1 (PSEN1) and Beta-Secretase 1 (BACE1) in the brain as well as decreased spatial memory. The addition of SAMe reduced the amyloid deposition, improved spatial memory, and decreased other damaging effects produced by the vitamin B deficiency. In a subsequent study [100], they found that the combination of SAMe and superoxide dismutase (SOD) have synergistic beneficial effects on all pathological brain parameters of Alzheimer disease in the TgCRND8 vitamin B-deficient mice. SAMe administration also protected brain cells and alleviated brain oxidative stress induced by amyloid-β, possibly by facilitation of the antioxidant defense mechanisms increasing the activities of glutathione peroxidase (GSH-Px), glutathione-S-transferase (GST), and SOD in the rat nervous system [101].

Several other studies demonstrated similar beneficial effects of SAMe on the progress of Alzheimer disease in animal models, which was associated with a reduction in the extent of oxidative stress in the affected brains and also in plasma thiol levels [102,103]. 

## 3. Valproic Acid-Basic Pharmacology

Valproic acid (VPA) is a branched-chained fatty acid derived from naturally occurring valproic acid that has antiepileptic activity among humans and various animals. It is also used for migraine, mood, and psychiatric disorders. Valproic acid acts through various mechanisms including an increase of gamma aminobutyric acid (GABA) in the brain due to the inhibition of its catabolism and reabsorption in the nervous system, the suppression of voltage sensitive channels [104,105,106,107], and the inhibition of histone deacetylase [108]. Johanessen et al. [107] showed a relationship between dose, brain levels, and a brain concentration-dependent effect in mice. The effects started with an increase in intracellular GABA and aspartate levels; then, with increasing brain levels, they found an increase in serotonin and monoamines, an inhibition of succinic semialdehyde dehydrogenase, and a decrease in ATP levels. The higher levels were associated with the inhibition of sodium currents, an increase in glutamate release, changes in the conductance of calcium channels, and the inhibition of GABA transaminase in the brain.

VPA can be used by oral, venous, and rectal routes. It is mostly bound to serum proteins. A decrease in protein binding is found in renal and hepatic diseases, at old age, during pregnancy, and with some other drugs (e.g., acetylsalicylic acid). The half-life in serum is 11–20 h [107]. It is mostly metabolized by the liver, and only a small fraction is excreted in the urine. The effective therapeutic plasma level is 50–100 microgram/mL^−1^. VPA has a low clearance, 6–20 mL/kg/hour due to the high serum protein binding. It is metabolized by glucuronidation in the liver microsomes, beta oxidation in the mitochondria, and oxidation in cytochrome P450. Valproate glucuronide is the main urine metabolite. Many genes with inter-individual variability affect its metabolism, and some genetic variants were associated with hepatic toxicity and teratogenicity. 

Zou et al. reviewed the major genetic variants that affect VPA metabolism, efficacy, and safety [109]. Variants associated with cytochrome P450 enzymes, uridine diphosphate glucuronyl-transferase gene variants (UGT) affected inter-individual pharmacokinetics. The polymorphism of several genes was associated with VPA hepatotoxicity. The FDA indicated contraindications for using VPA in patients with urea cycle disorders because of their risk of the development of hyperammonemia encephalopathy under VPA therapy, and some studies found an association of this phenomenon with variants of carbamoyl phosphate synthetase 1 (CPS1). Variants of polymerase gamma gene (POLG) and gluthatione S transferases (GSTs) were also associated with VPA liver toxicity. 

Since there is no correlation between VPA plasma concentration and antiepileptic effect and toxicity, Nakashima et al. [110] developed a population-based model based on 77 treated Japanese patients to determine an optimal personal therapeutic concentration. They evaluated the clinical characteristics, sodium channel neuronal type 1 alpha (SCN1A) polymorphism (which is associated in the Japanese population with carbamazepine resistant seizures with and without VPA), and the relation between VPA levels and seizures control. Patients were evaluated for age, seizure locus, seizure type, intellectual disability, and the co-administration of other antiepileptic drugs. They determined a model indicating an optimal concentration for each patient resulting in more than a 50% reduction in seizures frequency.

### Valproic Acid as a Histone Deacetylase Inhibitor (HDACI)

Histone deacetylase (HDAC) is a negative regulator of gene expression. VPA causes histone deacetylase inhibition [111,112], resulting in teratogenicity and cell toxicity. Phiel et al. [111] found that VPA at therapeutic levels mimicked the previously known HDACI Trichostatin A, causing the hyperacetylation of cultured cells, and the activation of transcription from exogenous and endogenous promotors. They also showed that both had teratogenic effects while other VPA analogous compounds that do not cause histone deacetylation inhibition and do not activate transcription are not teratogenic. VPA and its teratogenic analogues contained a tetrahedral alpha carbon bound to a free carboxyl group, a hydrogen group, and two alkyl groups, compared to the non-teratogenic analogues, which had no histone deacetylase inhibitory effect as they had another group added to the alpha carbon. 

Post-translational modification in the histone proteins that regulate gene expression controls about 10% of gene transcription. It is regulated by transferases that add or deacetylases that remove acetyl groups from the histone tail. Lysine acetylation in histones results in transcriptionally active genes, while lysine hypo-acetylation is associated with transcriptionally silent genes. Histone deacetylases impact important pathways (e.g., P53, nuclear factor kappa B (NFkB), hypoxia inducible factor alpha (HIF1α)). Disturbed HDAC activity was proven to be associated with cancer, neurodegenerative diseases, and metabolic, inflammatory, and cardiac diseases.

Sixto-Lopez et al. [113] studied the mechanism of HDACI activity of VPA. Out of the four HDAC classes, VPA is an inhibitor of the class I (HDAC 1, 2, 3, 8) and IIa (HDAC 4, 5, 7, 9). Analysis of the activity of VPA showed different inhibition profiles on the different HDACes. They found that VPA acted as a competitive inhibitor to HDAC 1, 2, 3 and 7 and as a non-competitive inhibitor to HDAC 4 and 6. They found that polar and non-polar energy favored the binding of HDAC 1, 2, 3, 7, and 8, while only polar energy favored the binding of HDAC 4 and 6. They concluded that the preferable selected inhibition of the different HDACes may explain the different potential effect on different disease states. 

An imbalance of enzymes from the HDAC class was associated with teratogenicity and autism. However, the inhibition of HDAC has a treatment potential for various stated diseases, including neurodegenerative diseases, the development of cancer, human immune deficiency virus (HIV), and more.

## 4. Hazardous Potential of VPA as HDACI

### 4.1. Teratogenicity

VPA is considered to be the most teratogenic antiepileptic drug inducing various malformations in animal models as well as in humans [104]. In humans, the more common malformations are neural tube defects (NTD), cardiac, limb, and skeletal anomalies, including oral clefts. Prenatal exposure to VPA also induces a variety of neurodevelopmental disorders including mental deficiency, learning difficulties, and autism [104,105]. 

We will not discuss this issue further, except for the issue related to neurodevelopmental disorders and to epigenetic changes resulting from the effects of VPA as an HDACI.

Chanda et al. used pluripotent human stem cells converted to reprogrammed neuronal cells to analyze VPA toxicity on neuronal maturation [114]. VPA exposure affected dendritic morphology and the neuronal function only of immature cells. Cells were treated with VPA on day 1, 21, and 50–56 after transgene induction, and they were evaluated after 72–96 h. VPA treatment on day 1 and 21 caused retardation in dendritic arborization in a dose-dependent manner with the worst outcome on day 1 for treated cells. On the other hand, the mature 60-day cells showed an increase in synapse size and number. Damage was associated with the inhibition of HDAC and glycogen synthase kinase-3 (GSK-3), which caused the down-regulation of genes including Macrophage Myristoylated Alanine-Rich C Kinase Substrate (MARCKSL1), an actin-stabilizing protein. 

### 4.2. VPA and Neurological Diseases

Autism: The transient hyperacetylation of histones H3 and H4 in the embryonic mouse brain was associated with autism. Katoka et al. [115] found that mice exposed to VPA on day 12.5 exhibited autistic-like behaviors. They found decreased numbers of cells in the middle and lower layers of the prefrontal cortex and in the lower layers of the somatosensory cortex. In addition, they observed an increase in apoptotic cell death in the neocortex and a decrease in cell proliferation in the ganglionic eminence. Moreover, an increase in acetylated histone H3 and H4 up to 6 hours from exposure was observed in whole tissue lysates of the embryonic brain. Moldrich et al. [116] exposed embryonic mice to VPA and to the HDAC inhibitor trichostatin A (TSA) on day 12.5 and showed one hour following treatment that both caused significant increases in histone H3 and H4 acetylation, and histone H3 lysine 4 tri-methylation. Both groups showed decreases in ultrasonic vocalization at postnatal day 8 (PND 8), olfactory motivation at PND 36, and elevated digging and grooming at PND 26–30, which was suggestive of mild restrictive and repetitive behaviors.

## 5. The Treating Potential of VPA as HDACI

### 5.1. Alzheimer Disease (AD)

Qing et al. [117] showed that VPA inhibited beta depositions and neuritic plaques in an APP23 transgenic AD mice model by inhibition of the gamma secretase cleavage of amyloid precursor protein and the inhibition of GSK-3 activity. Based on their findings, Noh et al. [118] evaluated VPA treatment in a Tg6779 murine AD model and found that VPA reduced the levels of neuroinflammatory cytokines at late symptomatic stages of AD by the inhibition of HDAC. A decrease in NFkB and IL-1 beta was found in the plasma and hippocampus and a decrease in TLR4 was found in the plasma. VPA increased the secreted form of amyloid precursor protein and the level of nerve growth factor (NGF) and its receptors in the hippocampus and the number of cholinergic neurons in the medial septum. Various other HDACI were also shown to reduce beta production and tau protein stabilization, making VPA a therapeutic candidate for AD patients.

### 5.2. Bipolar Disease

VPA is widely used to control psychiatric disorders, including bipolar disorder and manic psychosis. Besides increasing the levels of GABA and restraining neuronal repetitive firing, it is also suggested that the beneficial effects result from the direct inhibition of class I histone deacetylases. VPA was evaluated in a rat model of mania induced by dextroamphetamine (d-AMPH), which is a potent central nervous system dopaminergic stimulant. VPA reversed the d-AMPH-induced hyperactivity. The administration of VPA decreased the levels of histone deacetylase activity in the frontal cortex and striatum of the rats [119]. 

### 5.3. Cancer

The HDACI effect made VPA a candidate for cancer therapy. Hypermethylation and deacetylation of the CDK interacting protein/Kinase inhibitory protein (CIP/KIP) family were previously associated with tumorigenesis. Sanaei et al. [120] found that HDAI including VPA down-regulated DNA methyltransferase (DNMT1) and histone deacetylase 1 and up-regulated p21, p27, and p57 genes expression enhancing cell apoptosis and cell growth inhibition in human colon carcinoma cells in culture. The exposure of human neuroblastoma cells to VPA reduced the expression of the brain-derived neurotrophic factor (BDNF) receptor tropomyosin-related kinase receptor B (TrkB). VPA and the class 1 HDAC inhibitor entinostat similarly up-regulated RUNX3, a suppressor of TrkB gene that is highly expressed in aggressive neuroblastoma [119].

VPA was found to enhance the activity of other anti-cancer medications. When in combination with cisplatin, it potentiated its effects on non-small cell lung cancer cells by decreasing the ATP-binding cassette transporter 1 (ABCA1) activity through HDACII inhibition [121]. VPA induced cell death and autophagy in pancreatic cancer-derived cells by increasing the mitochondrial apoptotic pathway in the cancer cells. The VPA activity was in correlation with its ability to inhibit extracellular signal-regulated kinase (ERK) phosphorylation and mutant P53 expression. It also potentiated the effect of bortezomib, which similar to HDACI was shown to reduce NFkB activity and B-cell lymphoma-extra large (Bcl-xL) expression. Additionally, both affected the expression of the chaperone HSP70 [122]. 

### 5.4. HIV

Current HIV treatment reduce viral load and improve the immune function. However, despite improved CD4 cell counts and undetectable viral load, the HIV persists in latently infected CD4+ T cells. A strategy for eradication of the virus is to selectively reactivate CD4+ T cells, which will lead to viral replication, allowing susceptibility to combined immune and antiretroviral (ART) therapy. Since HDAC1 has a role in transcription and maintaining the latency of HIV after viral integration, VPA was offered to reverse its effect. Crosby et al. [123] reviewed the published clinical studies. They found that VPA did not have an appreciable efficacy as adjunctive therapy with combined antiretroviral treatment (ART) in promoting the reversal of viral latency. Despite few adverse effects only in one study, they indicate that the VPA treatment may lead to pharmacodynamic interactions with ART, such as thrombocytopenia, acute pancreatitis, and hepatotoxicity.

## 6. The Effects of SAMe in Pregnancy

Data on the long-term effects of SAMe and its safety for use during pregnancy are too limited to draw any definitive conclusions.

Only a few studies examined the effects of oral or intravenous SAMe treatment during pregnancy, and most of them focused on the efficiency of SAMe for the treatment of intrahepatic cholestasis of pregnancy (obstetric cholestasis), which is a common liver disorder specific to pregnancy that is characterized by maternal pruritus and increased serum bile acid concentrations [Frezza, 1990 #90;Ribalta, 1991 #91;Zhang, 2015 #100;Burrows, 2001 #102;Nicastri, 1998 #103;Frezza, 1984 #104;Guo, 2015 #324]. Most of these studies did not report neonatal outcome except for preterm labor and APGAR score. Infant’s follow up was described only by Riblata et al. [124], who reported normal postnatal development up to 3 months of age with no follow-up later. In these studies, SAMe was given during the third trimester of pregnancy. Thus, the safety of SAMe administration was not examined during the organogenesis stage and despite SAMe’s appearance as safe, it is not known whether supplementation during pregnancy affects the risk of adverse pregnancy outcome. Hence, trials of SAMe during pregnancy and breast-feeding with long-term monitoring of child development would be important.

Animal studies: There are relatively few animal studies on the safety of SAMe in pregnancy. Most of them used SAMe in combination with other substances. Ubeda-Martín et al. [125] found that the co-administration of VPA (300 mg/kg/day on gestational days 8–10) and SAMe (10 mg/kg/day, on gestational days 1–10) in rats did not reduce the incidence of VPA-associated neural tube defects in the offspring. 

Seyoum et al. [126] treated 9.5-day-old cultured rat embryos with ethanol, SAMe, or a combination of both. They found that SAMe administration prevented ethanol’s developmental toxicity. The administration of SAMe alone did not affect embryonic development.

## 7. Effects of SAMe and VPA in Pregnancy

There are only a few studies on the possible effects of SAMe or other methyl donors in combination with VPA in pregnancy. Ubeda-Martin et al. [125] treated pregnant rats with VPA on days 8–10 of pregnancy or VPA and SAMe, the latter being given daily during the first 10 days of gestation. They found no protective effects of SAMe on the VPA-induced fetal damage. 

In spite of the clinical advice to antiepileptic drug-treated epileptic women to take high doses of folic acid before and during pregnancy, there seems to be insufficient data to support the benefits of high doses of folic acid for the reduction of NTD or other malformations induced by VPA or carbamazepine [127]. Nor is there sufficient proof for the beneficial effect of folic acid in the prevention of VPA-induced ASD [128]. Recently, Turgut et al. [129] treated chick embryos with VPA, producing a high rate of NTD. The concomitant administration of high doses of folic acid only slightly and insignificantly reduced the rate of NTD in the embryos. 

In our previous studies, we treated newborn ICR albino mice on postnatal day 4 with a single injection of 300 mg/kg of VPA, with normal saline (controls), or with SAMe that was given orally for 3 days at a dose of 30 mg/kg body weight. Other animals were treated with a combination of VPA and SAMe. From day 50, we carried out neurobehavioral tests for 10 days. On day 60, the animals were euthanized, and the prefrontal cortex and liver were removed for biochemical and molecular studies. We found that VPA caused ASD-like neurobehavioral deficits, which were more prominent in males compared to females. In addition, several markers of oxidative stress such as lipid peroxidation, SOD and CAT activity and their gene expression were increased in the brain but not in the liver, demonstrating increased brain oxidative stress as late as the age of 60 days! VPA also induced sex-related significant changes in the expression of many neurophysiology and neuropathology genes in the prefrontal cortex, as demonstrated by Nanostring nCounter analysis. SAMe alone induced changes in the expression of only a few genes. SAMe administration to the VPA-treated mice alleviated most ASD-like neurobehavioral symptoms, reduced the degree of oxidative stress in the prefrontal cortex, and normalized most of the VPA-induced gene expression changes [130].

In a subsequent recent study, we treated pregnant ICR albino mice on day 12 of gestation with a single dose of 600 mg/kg of VPA or with normal saline. Each of these two groups was further subdivided into two groups: one group received 30 mg/Kg of SAMe by intraoral gavage once daily for 3 days starting on the day of VPA injection, and the other group similarly received phosphate-buffered saline. The offspring were euthanized on PND 1. The rostral half of the brain, including cerebral hemispheres and hippocampi, were removed for biochemical and molecular studies similar to the above described investigations. We found that SAMe but not VPA treatment induced multiple and significant gene expression changes in the frontal part of the brain, with sex-specific differences. In addition, SAMe treatment also increased brain oxidative stress affecting lipid peroxidation, SOD, and catalase (CAT) activity. Co-administration of VPA with SAMe normalized almost all gene expression changes produced by SAMe alone. Thus, an antagonistic action (interaction) between SAMe and VPA was demonstrated with prenatal treatment as well as with early postnatal treatment [131]. Interestingly, the main pathway affected by SAMe administration was the Vegfa (vascular endothelial growth factor a) pathway with 13 out of 26 genes significantly changed in males and 9 out of 26 genes in the Vegfa pathway changed in females. In males, the z score of the VEGF pathway was statistically significant, meaning that the pathway was up-regulated as predicted; in females, the changes in the expression of Vegf genes pathway did not have a significant direction as genes were either up or down-regulated. The most affected genes in both genders were Vegfa and its receptor Flt1 (Fms Related Receptor Tyrosine Kinase 1) up-regulated by 390% and 219% in males, and 272% and 292% in females, respectively. Vegf pathway overexpression was reported to be involved in cognitive decline and neurological alternation in adults [132,133,134,135] as well as behavioral changes in rodents [136]. 

Our results demonstrate that the in utero developmental period serves as a critical time window during which maternal exposure to methyl-donors supplementation can influence gene expression in the brain. Such epigenetic changes induced by SAMe during the late embryonic and early fetal periods are not induced if SAMe is administered during the first week of postnatal life, demonstrating that there is a limited time in the mouse fetus when methyl donors can affect the epigenome. 

Interestingly, one may predict that the extensive changes in gene expression induced by prenatal SAMe in the mouse fetuses will significantly affect fetal viability, causing congenital malformations and postnatal deviations in weight gain and viability. We followed the offspring for one month and found 100% viability, no external malformations, and normal growth in comparison to controls. Moreover, we also found no differences in the developmental analysis between the SAMe-treated and control groups, including eye opening, surface righting, cliff aversion, rooting, forelimb grasp, auditory startle, ear twitch, and open field traversal tests [137]. 

Thus, in spite of the increased brain oxidative stress and abnormalities in gene expression, the long-term negative effects seem to be minimal. This raises the possibility that some of the epigenetic changes induced by SAMe are reversed postnatally, apparently resulting from the influence of the postnatal environment that was similar in the SAMe-treated and control groups. Additional studies are needed to further clarify this issue.

The prevention/amelioration of the postnatal VPA-induced changes in gene expression by SAMe and the prevention of the prenatal SAMe-induced changes in gene expression by VPA point to the antagonistic effects of these agents. SAMe is an important methyl donor, and VPA with its histone deacetylase inhibitory action increases DNA demethylation. The mechanisms underlying these antagonistic effects are as yet unknown. The fact that each one, SAMe and VPA, prevent/ameliorate the abnormalities induced by the other agent point to the need to further explore other therapeutic possibilities using the combination of these agents.

We did not find additional studies on the possible effects of SAMe in pregnant rodents on gene expression in the offspring. However, there are studies on other methyl donors. Studies in rodents have shown that both maternal and paternal supplemental intake of methyl-group donors before conception and/or maternal intake during pregnancy can influence fetal methylation patterns, gene expression, metabolic status, and behavior in adult offspring [138]. The offspring of mice fed a methyl-supplemented diet during pregnancy have shown a phenotypic shift toward brown coat color and alerted feeding behavior [139]. The offspring of dams fed diets supplemented by high doses of folic acid during pregnancy displayed short-term memory impairment, decreased hippocampal size, decreased thickness of the dentate nucleus at 3 weeks of age, and an altered development of the cortical layers at E17.5 [140]. Thus, these studies point to the possibility of long-term neurobehavioral damage caused by high doses of methyl donors. Whether SAMe may produce similar damage awaits future studies.

## 8. Conclusions

SAMe and VPA are both epigenetic modifiers with apparent antagonistic effects on DNA methylation. Their possible use for the treatment of mental and neurological disorders that exhibit epigenetic modifications are so far exploratory. While there is much data regarding the effects of VPA in pregnancy, less is known about the possible effects of SAMe in pregnancy. Both substances seem to have different and possibly antagonistic effects on DNA methylation. Indeed, at least in mice, they seem to antagonize each other, as early postnatal VPA will induce in mice autistic-like behavior and significant, sex-related, modifications in the gene expression of the brain. These effects are ameliorated or even prevented by the concomitant administration of SAMe. In contrast, prenatal administration of SAMe in mice induced sex-related changes in gene expression of the brain that were prevented/ameliorated by VPA. There seems to be an urgent need for additional studies in order to elucidate the mechanism/s of these antagonistic effects and explore additional possibilities for successful use of these substances in a variety of diseases that stem, even partially, from epigenetic modifications. 

## Abbreviations

ASD	Autism spectrum disorder
3-MHPG	3-Methoxy-4-hydroxyphenylglycol
5caC	5-carboxyl-cytosine
5fC	5-formyl-cytosine
5HIAA	5-hydroxyindoleacetic acid
5hmC	5-hydroxymethylcytosine
5mC	5methylcytosine
MECP2	Methyl CPG binding protein 2
ART	Antiretroviral therapy
ABCA1	ATP-binding cassette transporter 1
BACE1	Beta-Secretase 1
BDNF	Brain-derived neurotrophic factor
CPS1	Carbamoyl phosphate synthetase 1
CAT	Catalase
CNS	Central nervous system
CSF	Cerebrospinal fluid
CP	Critical period
d-AMPH	Dextroamphetamine
DNMTs	DNA methyltransferases
ERK	Extracellular signal-regulated kinase
Flt1	Fms Related Receptor Tyrosine Kinase 1
FST	Forced swim test
GABA	Gamma aminobutyric acid
GD	Gestational Day
UGT	Glucuronyl-transferase
GSH	Glutathione
GSH-Px	Glutathione peroxidase
GST	Glutathione-S-transferase
GSK-3	Glycogen synthase kinase-3
HAT	Histone acetyltransferases
HDACs	Histone deacetylases
HCY	Homocysteine
HVA	Homovanillic acid
HIV	Human immune deficiency virus
HPG axis	Hypothalamic–pituitary–gonadal axis
HIF1α	Hypoxia inducible factor alpha
IEG	Immediate–early gene
LSD	Lysine-specific demethylase
MARCKSL1	Macrophage Myristoylated Alanine-Rich C Kinase Substrate
MDD	Major depression disorder
MBD	Methyl-binding domain
NGF	Nerve growth factor
NTD	Neural tube defects
NMDA	N-Methyl-D-Aspartate
NFkB	Nuclear factor kappa B
PLOG	Polymerase gamma gene
PSMS	Post-translational histone modifications
PSEN1	Presenilin-1
RNS	Reactive nitrogen species
ROS	Reactive oxygen species
SAH	S-adenosylhomocysteine
SAMe	S-adenosylmethionine
SAHH	SAH hydrolase
SCN1A	Sodium channel neuronal type 1 alpha
SOD	Superoxide dismutase
TET	Ten-eleven translocation
TdT	Terminal deoxynucleotidyl transferase
Thcy	Total HCY
TSA	Trichostatin A
TrkB	Tropomyosin-related kinase receptor B
VPA	Valproic acid
Vegf-a	Vascular endothelial growth factor a
